# Efficacy of *Ginkgo biloba* extract in amyloid PET-positive patients with mild cognitive impairment

**DOI:** 10.3389/fneur.2025.1639924

**Published:** 2025-08-15

**Authors:** YoungSoon Yang, Min-Seong Koo, Yong Tae Kwak

**Affiliations:** ^1^Department of Neurology, Soonchunhyang University Cheonan Hospital, Cheonan-si, Chungcheongnam-do, Republic of Korea; ^2^Department of Psychiatry, International St. Mary's Hospital, Catholic Kwandong University College of Medicine, Seo-gu, Incheon, Republic of Korea; ^3^Department of Neurology, Hyoja Geriatric Hospital, Kuseong-myeon, Yongin-si, Gyeonggi-do, Republic of Korea

**Keywords:** *Ginkgo biloba*, mild cognitive impairment, Alzheimer’s disease, amyloid PET, MDS-Oaβ

## Abstract

**Background:**

Mild cognitive impairment (MCI) with amyloid PET positivity represents a prodromal stage of Alzheimer’s disease (AD), yet no disease-modifying therapies are currently approved. *Ginkgo biloba*, traditionally used in East Asian and European ethnomedicine as an oral decoction or standardized extract to support memory and cognitive function, is commonly utilized, however, its efficacy as monotherapy in biomarker-confirmed MCI remains uncertain. Aβ oligomers, produced by abnormal cleavage of amyloid precursor protein, disrupt synaptic function and contribute to cognitive decline.

**Objective:**

This study evaluated whether *Ginkgo biloba* alone, without adjunctive anti-dementia medication, could provide clinical and biomarker benefits in amyloid PET–positive MCI patients. Plasma MDS-Oaβ (Multimer Detection System–Oligomeric Aβ), a dynamic biomarker reflecting Aβ oligomerization tendency, was used to explore mechanistic relevance.

**Methods:**

In this retrospective cohort study, 64 amyloid PET–positive MCI patients were followed for 12 months. Participants received either oral *Ginkgo biloba* monotherapy (240 mg/day, *n* = 42) or standard cognitive enhancers (*n* = 22). Clinical outcomes included the Korean version of the Mini-Mental State Examination (K-MMSE), Clinical Dementia Rating–Sum of Boxes (CDR-SB), Korean Instrumental Activities of Daily Living (K-IADL), and Neuropsychiatric Inventory (NPI). Plasma MDS-Oaβ levels were assessed at baseline and at 12 months.

**Results:**

At 12 months, the Ginkgo group showed significantly higher responder rates (100% vs. 59.1%, *p* < 0.001), no conversion to AD dementia (0% vs. 13.6%, *p* = 0.037), and greater improvement in K-MMSE and K-IADL scores. MDS-Oaβ levels decreased significantly in the Ginkgo group (*p* < 0.001) but not in the control group. No significant between-group differences were observed in CDR-SB or NPI scores.

**Conclusion:**

*Ginkgo biloba* monotherapy was associated with preserved cognition, improved daily functioning, and reduced plasma Aβ oligomerization in amyloid PET–positive MCI patients. These findings suggest potential disease-modifying effects and warrant further validation in prospective, biomarker-based clinical trials.

## Introduction

Alzheimer’s disease (AD) is the most common cause of dementia, exerting a substantial global burden. Although the pathological roles of amyloid-beta (Aβ) aggregation and tau have been increasingly clarified, translating these insights into effective treatments remains a major challenge (1). Mild cognitive impairment (MCI) is a transitional stage between normal aging and dementia, and individuals with amyloid PET–positive MCI are at particularly high risk of progressing to AD. Previous studies have characterized amyloid PET–positive MCI as prodromal AD (2, 3), with subtle impairments in memory and executive function that preserve functional independence. The annual conversion rate to AD among biomarker-confirmed MCI patients is estimated at 5–30% (4, 5). However, no disease-modifying treatments are currently approved for MCI. In South Korea and many other countries, standard anti-dementia drugs like donepezil, rivastigmine, galantamine, and memantine are not reimbursed for MCI (6), leading to widespread off-label use of cognitive supplements such as omega-3 fatty acids, choline precursors, and *Ginkgo biloba*.

*Ginkgo biloba* leaves have been traditionally utilized in East Asian ethnomedicine, particularly within Chinese and Korean medical traditions, as an oral decoction or herbal remedy to enhance memory, mitigate age-related cognitive decline, and alleviate circulation-related symptoms such as dizziness and tinnitus (7). Currently, standardized *Ginkgo biloba* extracts are widely employed both as prescription phytomedicine in Europe and as a non-prescription cognitive supplement globally. Despite their broad usage, the efficacy of *Ginkgo biloba* extracts as monotherapy in biomarker-confirmed mild cognitive impairment (MCI) remains uncertain.

At our institution, *Ginkgo biloba* extract has been occasionally prescribed depending on clinical context, and our accumulated clinical experience prompted a more systematic investigation. We employed Ginexin-F^®^, a standardized extract approved by the Korean Ministry of Food and Drug Safety (MFDS-199702183). Its composition is equivalent to EGb 761, a widely studied formulation in clinical trials. Ginkgo’s neuroprotective effects have been attributed to antioxidative, anti-inflammatory, and vasodilatory properties, as well as modulation of neurotransmission and inhibition of Aβ aggregation (7–9). Although many studies have examined Ginkgo’s effects in MCI, results remain mixed. A placebo-controlled trial showed cognitive benefits with good adherence (10), whereas the large GEM trial in cognitively normal elderly individuals failed to show preventive effects, likely due to low incidence of dementia and poor compliance (11). The GUIDAGE trial in patients with subjective memory complaints also yielded inconclusive results (12, 13). Early meta-analyses indicated modest cognitive improvements (14), but a Cochrane review in 2007 questioned their robustness (15). More recent randomized trials and meta-analyses suggest EGb 761 may offer symptomatic benefit in MCI. One 12-month trial in China reported reduced dementia incidence in amnestic MCI patients treated with Ginkgo (16), while others noted improvements in memory and neuropsychiatric symptoms (17, 18). Still, a recent systematic review concluded that definitive evidence for Ginkgo in MCI remains insufficient (19).

This inconsistency likely reflects the heterogeneity of MCI itself. While some patients harbor prodromal AD pathology, others may have non-AD causes of cognitive symptoms. This variability complicates trials, as AD-targeting therapies may be tested on non-AD cases. Furthermore, the slow progression of early-stage AD challenges the sensitivity of conventional outcome measures. To address these issues, we integrated biomarker-based strategies for diagnosis and treatment monitoring. Our group previously reported a retrospective study combining donepezil and Ginkgo in amyloid PET–positive AD patients, using plasma MDS-Oaβ (Multimer Detection System–Oligomeric Aβ) as a biomarker (20). This blood-based assay measures the oligomerization tendency of Aβ and has shown associations with cognitive scores, disease progression, and CSF tau levels (21). In that study, the combination group demonstrated improved cognition and greater reductions in MDS-Oaβ. However, all patients received donepezil, limiting evaluation of Ginkgo’s independent effect. Because anti-dementia drugs are not reimbursed for MCI in Korea, *Ginkgo biloba*—being accessible and affordable—is often used as a practical alternative due to its accessibility and affordability. This creates a real-world setting to examine its effects without confounding treatments.

In this study, we focused on amyloid PET–positive MCI patients to enhance diagnostic specificity. We evaluated cognitive and functional changes as well as plasma MDS-Oaβ over 12 months. Unlike static biomarkers such as Aβ42 or total Aβ, MDS-Oaβ reflects the dynamic propensity for toxic oligomer formation, a key pathogenic step in AD (21). This study was designed to assess whether *Ginkgo biloba* monotherapy could provide both symptomatic and biomarker-level benefits in patients with amyloid-confirmed MCI. To this end, we analyzed real-world registry data and applied validated biomarker assays to evaluate its potential as a practical early intervention.

## Materials and methods

### Study design

This retrospective cohort study utilized data from the Soonchunhyang Dementia Registry, a longitudinal database capturing clinical, cognitive, and biomarker data from patients evaluated at the Dementia Clinic of Soonchunhyang University Cheonan Hospital. The registry includes detailed diagnostic evaluations and serial follow-ups since March 2020, offering a complete medical history, physical and neurological examinations, comprehensive neuropsychological testing, and routine laboratory tests including ApoE. Magnetic resonance imaging (MRI) is performed within 3 months, and ^18^F-FC119S PET/computed tomography (CT) is performed when possible in the same period.

### Participants

Participants were eligible if they met the following criteria: (1) diagnosis of MCI according to Petersen et al. (22) criteria, which includes subjective cognitive complaints corroborated by an informant, objective impairment in one or more cognitive domains (typically memory), preserved global cognitive function, largely intact activities of daily living, and absence of dementia; (2) evidence of cerebral amyloid pathology confirmed by ^18^F-FC119S PET (3) no prior use of anti-dementia medications including cholinesterase inhibitors or memantine; (4) a minimum follow-up duration of 12 months; and (5) available plasma samples at baseline and 12 months for MDS-Oaβ analysis. Patients were excluded if they had major psychiatric illness, stroke, or other neurological conditions that could confound cognitive assessments.

### Group allocation and treatments

Patients were categorized into two treatment groups based on their initial post-diagnostic management. The Ginkgo group received 240 mg/day of a standardized *Ginkgo biloba*. The non-Ginkgo group received other commonly used cognitive enhancers, including omega-3 fatty acid supplements (with ≥600 mg DHA + EPA combined daily) or choline precursors. Importantly, no patients in either group received prescription anti-dementia drugs during the study period. Treatment decisions were made by the managing neurologist based on clinical judgment, patient preference, and insurance coverage limitations.

### Cognitive and functional assessments

Neuropsychological testing included the Korean version of the Mini-Mental State Examination (K-MMSE) to evaluate global cognitive function, and the Clinical Dementia Rating-Sum of Boxes (CDR-SB) to assess multidomain cognitive and functional abilities. Functional status was further assessed with the Korean Instrumental Activities of Daily Living (K-IADL) scale, with a K-IADL score (Sum of item scores/Number of items answered) of ≥0.40 indicating probable conversion to dementia (23). Behavioral symptoms were evaluated using the Neuropsychiatric Inventory (NPI), which covers 12 domains including depression, apathy, agitation, and hallucinations. All assessments were conducted at baseline and repeated after approximately 12 months. Due to the observational nature of the study, slight variations in follow-up timing were allowed, but all reassessments occurred within a 10–14 month window.

### Definition of clinical response

To assess treatment response, we applied the “no deterioration” criterion, commonly used in real-world studies. Patients were classified as responders if there was no decline in K-MMSE and no increase in CDR-SB over the 12-month period. Those who showed any decline in K-MMSE or any increase in CDR-SB during the same period were not classified as responders. Patients were considered to have converted to AD if their K-IADL score increased from <0.40 at baseline to ≥0.40 at 12-month follow-up, based on validated Korean criteria (23).

### Safety monitoring

Adverse events were monitored at each clinic visit by asking patients and caregivers about new symptoms and through direct clinical observation. Events were recorded and categorized by severity. Discontinuation due to side effects was also noted.

### Plasma biomarker measurement

MDS-Oa*β* was used to quantify the amyloid-β oligomerization tendency in plasma. This assay measures how readily monomeric Aβ peptides form soluble toxic oligomers under standardized conditions. At each measurement point, plasma samples were thawed and incubated with synthetic Aβ peptides for 48 h at 37°C. Following incubation, samples were treated with a chemiluminescent substrate, and oligomerization levels were quantified using a Victor 3 luminometer. To ensure analytical reliability of the MDS-Oaβ assay, synthetic Aβ42 peptide was utilized as an internal standard, as previously described (24, 25). Reagents, including synthetic Aβ peptides and capture antibodies, were consistently obtained from the same manufacturing lot to minimize lot-to-lot variability. Assay reproducibility was routinely monitored by assessing intra- and inter-assay coefficients of variation (CV), which were maintained below 10%. Plasma samples underwent a single freeze–thaw cycle; specifically, samples were thawed at 37°C for 15 min immediately prior to analysis, as previously validated (25, 26). All samples were analyzed concurrently to avoid inter-batch variability. The test has been validated in prior studies to correlate with CSF tau levels, cognitive scores, and PET findings, providing a dynamic and sensitive readout of early amyloid pathology (21, 24, 25, 27).

### Statistical analysis

Continuous variables were compared between groups using independent *t*-tests, and within-group changes were analyzed with paired *t*-tests. Categorical variables, including response status and conversion rates, were assessed using the chi-square test or Fisher’s exact test, as appropriate based on cell counts. All statistical analyses were performed using SPSS version 24.0 (IBM Corp., Armonk, NY), and a *p*-value < 0.05 was considered statistically significant.

## Results

### Baseline demographic and clinical characteristics

**Figure 1:**
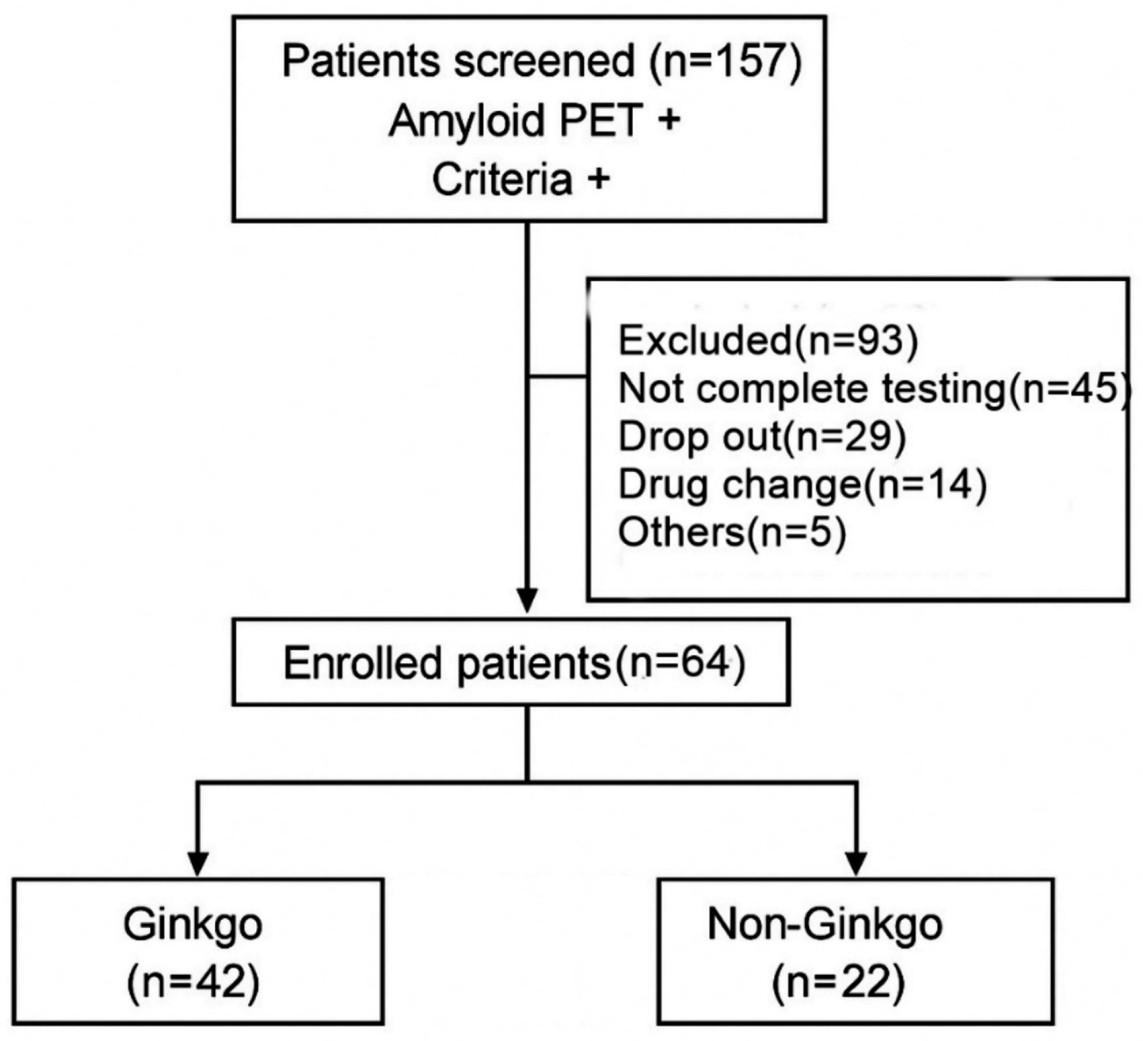
Flow chart of patients eligible for the study.

A total of 157 patients who were amyloid PET positive and met inclusion criteria were screened. Among them, 93 patients were excluded due to incomplete testing (*n* = 45), dropout (*n* = 29), drug changes (*n* = 14), or other reasons (*n* = 5). Finally, 64 patients were enrolled, with 42 patients in the Ginkgo group and 22 in the Non-Ginkgo group (Figure 1). There were no significant differences between groups in age (65.8 vs. 68.6 years), sex distribution (73.8% vs. 81.8% female), education (12.6 vs. 11.1 years), or ApoE4 allele count (0.62 vs. 0.73). Baseline cognitive and functional measures, including K-MMSE (28.4 vs. 28.5), CDR (both 0.5), CDR-SB (0.8 vs. 0.9), and K-IADL (3.4 vs. 3.0), were similar between groups. Neuropsychiatric symptoms (NPI) and plasma MDS-Oaβ levels (0.88 vs. 0.89 ng/mL) also showed no significant differences (Table 1).

**Table 1:** Baseline demographics and clinical variables of study subjects.

Variables	Ginkgo (*n* = 42)	Non-Ginkgo (*n* = 22)	*p*-value^*^
Age, years	65.8 ± 10.4	68.6 ± 7.9	0.288
Female gender (%)	31(73.8%)	18(81.8%)	0.348
Education years	12.6 ± 4.7	11.1 ± 4.9	0.244
ApoE4 gene number	0.62 ± 0.73	0.73 ± 0.83	0.593
K-MMSE	28.4 ± 1.2	28.5 ± 1.2	0.763
CDR	0.5 ± 0.0	0.5 ± 0.0	NA
CDR-SB	0.8 ± 0.5	0.9 ± 0.5	0.894
K-IADL	3.4 ± 2.8	3.0 ± 1.4	0.477
NPI	3.7 ± 5.1	1.2 ± 3.3	0.149
MDS-Oaβ (ng/mL)	0.88 ± 0.15	0.89 ± 0.11	0.838

^*^Between-group comparisons were conducted using independent *t*-tests. K-MMSE, Korean Mini-Mental State Examination; CDR, Clinical Dementia Rating Scale; CDR-SB, Clinical Dementia Rating Scale Sum of Box; K-IADL, Korean Instrumental Activities of Daily Living; NPI, Neuropsychiatric Inventory; MDS-Oaβ, Multimer Detection System-Oligomerized Aβ assay; NA, not applicable due to zero variance.

### Cognitive and functional response at 12 months

At the 12-month follow-up, all 42 patients in the Ginkgo group maintained stable cognitive status according to the responder criteria (no decline in K-MMSE and no increase in CDR-SB). In contrast, only 13 of the 22 patients (59.1%) in the non-Ginkgo group met the responder criteria (*p* < 0.001). Conversion to Alzheimer’s disease was defined by a K-IADL score ≥ 0.40 at 12 months. No patients in the Ginkgo group met this criterion at 12 months, while three patients (13.6%) in the non-Ginkgo group did (*p* = 0.037), indicating a statistically significant difference in functional outcomes between the groups (Table 2).

**Table 2:** Clinical response at 12 months in Ginkgo and non-Ginkgo groups.

Outcome	Ginkgo	Non-Ginkgo	*p*-value
Stable cognitive status (responder)	42(100.0%)	13(59.1%)	0.000
Cognitive decline (non-responder)	0(0.0%)	9(40.9%)	
No conversion to AD (K-IADL < 0.40)	42(100.0%)	19(86.4%)	0.037
Conversion to AD (K-IADL ≥ 0.40)	0(0.0%)	3(13.6%)	

Stable cognitive status (Responder) was defined as no decline in K-MMSE and no increase in CDR-SB scores over 12 months. Conversion to Alzheimer’s disease (AD) was defined as achieving a Korean Instrumental Activities of Daily Living (K-IADL) score ≥ 0.43 at 12 months. Values are number of patients (percentage within group). Statistical significance between groups was assessed using the chi-square test.

### Change in cognitive and biomarker outcomes over 12 months

**Figure 2:**
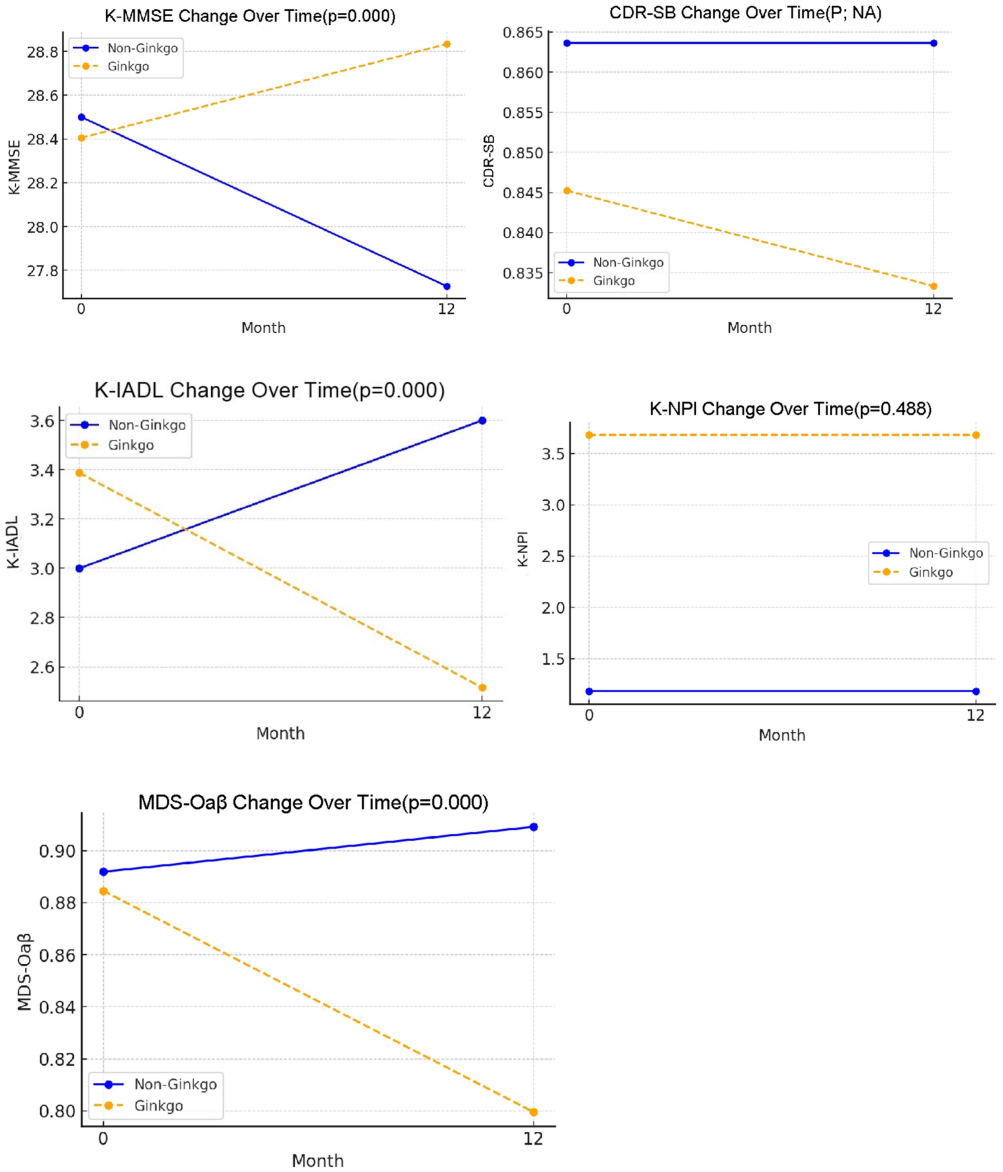
Change of K-MMSE, CDR-SB, K-IADL, K-NPI, and MDS-Oaβ in mild cognitive impairment patients with Ginko and Non-Ginko treatment.

Table 3 summarizes the changes in cognitive and biomarker outcomes from baseline to 12 months. K-MMSE scores increased slightly in the Ginkgo group (28.4 vs. 28.8), reflecting an average gain of 0.4 points (*p* = 0.008, within-group). In contrast, the non-Ginkgo group exhibited a mean decline of 0.8 points (*p* = 0.008, within-group). The between-group difference in MMSE change was statistically significant (*p* < 0.001). CDR-SB scores remained unchanged in both groups, averaging 0.8 in the Ginkgo group and 0.9 in the non-Ginkgo group at both timepoints. K-IADL total scores improved in the Ginkgo group from 3.4 to 2.5 (Δ − 0.8, *p* = 0.001), while they worsened in the non-Ginkgo group from 3.0 to 3.5 (Δ + 0.6 ± 0.9, *p* = 0.013). This between-group difference was statistically significant (*p* < 0.001), indicating a meaningful functional benefit of Ginkgo therapy. There were no significant changes in NPI scores in either group over 12 months. Plasma MDS-Oaβ levels significantly decreased in the Ginkgo group, from 0.88 to 0.80 ng/mL, corresponding to a mean reduction of 0.08 ng/mL (*p* < 0.001, within-group). Conversely, the non-Ginkgo group exhibited a non-significant increase from 0.89 to 0.91 ng/mL (Δ + 0.02; *p* = 0.255). The between-group difference in MDS-Oaβ change was statistically significant (*p* < 0.001) (Figure 2).

**Table 3:** Comparison of clinical outcome and MDS-Oaβ between Ginkgo and non-Ginkgo groups over 12 months.

Measure	Group	0 month	12 month	delta	P1^*^	P2^*^
K-MMSE	Ginkgo	28.4 ± 1.2	28.8 ± 0.9	0.4 ± 0.9	0.008	0.000
	Non-Ginkgo	28.5 ± 1.2	27.7 ± 0.9	−0.8 ± 1.2	0.008	
CDR-SB	Ginkgo	0.8 ± 0.5	0.8 ± 0.5	0.0 ± 0.1	0.323	NA
	Non-Ginkgo	0.9 ± 0.5	0.9 ± 0.5	0.0 ± 0.0	NA	
K-IADL	Ginkgo	3.4 ± 2.8	2.5 ± 2.7	−0.8 ± 1.3	0.001	0.000
	Non-Ginkgo	3.0 ± 1.4	3.5 ± 1.4	0.6 ± 0.9	0.013	
NPI	Ginkgo	3.7 ± 5.1	3.7 ± 5.1	0.0 ± 0.1	0.329	0.488
	Non-Ginkgo	1.2 ± 3.3	1.2 ± 3.5	0.0 ± 0.0	0.457	
MDS-Oaβ (ng/mL)	Ginkgo	0.88 ± 0.15	0.80 ± 0.11	−0.09 ± 0.10	0.000	0.000
	Non-Ginkgo	0.89 ± 0.11	0.91 ± 0.15	0.02 ± 0.07	0.255	

*Within-group comparisons (P1) were conducted using paired *t*-tests, and between-group comparisons (P2) were conducted using independent *t*-tests. P1, Baseline vs Follow-up *p*-value (Within-group); P2, Ginkgo vs. non-Ginkgo *p*-value (Between-group); K-MMSE, Korean Mini-Mental State Examination; CDR, Clinical Dementia Rating Scale; CDR-SB, Clinical Dementia Rating Scale Sum of Box; K-IADL, Korean Instrumental Activities of Daily Living; NPI, Neuropsychiatric Inventory; MDS-Oaβ, Multimer Detection System-Oligomerized Aβ assay, NA, Not applicable due to zero variance. Due to zero variance in the Non-Ginkgo group, statistical comparison was not applicable (NA). Hence, no conclusion can be drawn regarding group differences.

### Analyzing the influencing variable for responder

In a linear regression model predicting responder status at 12 months, treatment group (Ginkgo vs. non-Ginkgo) was the only significant predictor (*B* = 0.319, SE = 0.111, standardized β = 0.524, *t* = 2.870, *p* = 0.009). No other variables—including sex, age, education, baseline MDS-Oaβ, CDR-SB, K-MMSE, K-IADL NPI, ApoE4 allele—were significantly associated with responder status (all *p* > 0.10) (Table 4).

**Table 4:** Linear regression analysis predicting responder status at 12 months.

Predictor	B	SE	Beta	*t*	*p*-value
Constant	−1.466	1.587	–	−0.924	0.366
Sex (male, female)	0.006	0.117	0.011	0.056	0.956
Age (years)	−0.007	0.007	−0.228	−1.002	0.327
Education (years)	0.002	0.014	0.029	0.138	0.892
MDS-Oaβ	0.379	0.445	0.168	0.852	0.403
CDR-SB	−0.065	0.111	−0.134	−0.588	0.562
Baseline MMSE	0.083	0.057	0.305	1.443	0.163
NPI	0.021	0.013	0.342	1.709	0.102
Number of ApoE4	−0.030	0.097	−0.079	−0.306	0.762
K-IADL	−0.006	0.021	−0.056	−0.284	0.779
Group (Ginkgo vs. non-Ginkgo)	0.319	0.111	0.524	2.870	0.009

Linear regression was conducted to identify predictors of responder status, defined as the absence of decline in K-MMSE and no increase in CDR-SB over a 12-month period. Independent variables included demographic, clinical, and biomarker-related factors. A significant association was found between Ginkgo group membership and responder status (*p* = 0.009). K-MMSE, Korean Mini-Mental State Examination; CDR, Clinical Dementia Rating Scale; CDR-SB, Clinical Dementia Rating Scale Sum of Box; K-IADL, Korean Instrumental Activities of Daily Living; NPI, Neuropsychiatric Inventory; MDS-Oaβ, Multimer Detection System-Oligomerized Aβ assay.

### Adverse events

Adverse events were generally mild and transient in both groups. In the Ginkgo group, the most commonly reported events were diarrhea (7.1%), headache (4.8%), nausea (4.8%), and dizziness (4.8%). The non-Ginkgo group experienced similar side effects, including nausea (9.0%), diarrhea (9.0%), and headache (4.5%). One case of skin rash occurred in the Ginkgo group. Importantly, no patient in either group discontinued treatment due to adverse events, suggesting favorable tolerability (Table 5).

**Table 5:** Adverse events in follow up study group.

Adverse event	Ginkgo (*n* = 42)	Non-Ginkgo (*n* = 22)
Diarrhea	3(7.1%)	2(9.0%)
Headache	2(4.8%)	1(4.5%)
Nausea	2(4.8%)	2(9.0%)
Vomiting	1(2.4%)	1(4.5%)
Dizziness	2(4.8%)	0(0.0%)
Skin lesion	1(2.4%)	0(0.0%)

In the Ginkgo group, 3 patients (7.1%) had multiple adverse events, and in the Non-Ginkgo group, 1 patients (4.5%) had multiple adverse events.

## Discussion

This retrospective cohort study aimed to evaluate the efficacy of *Ginkgo biloba* monotherapy in patients with amyloid PET–positive MCI, using both clinical and biomarker endpoints. Our findings suggest that Ginkgo, when used alone without concomitant prescription anti-dementia medications, may offer measurable benefits in terms of cognitive stability, functional maintenance, and reduction in plasma MDS-Oaβ levels over a 12-month follow-up period. In multivariate linear regression with responder status as the dependent variable, only Ginkgo group was significantly associated with being a responder, no other covariates reached statistical significance (Table 4). Our results are consistent with findings from recent studies that explored the use of EGb 761 in MCI and early dementia. For example, Tian et al. demonstrated a reduced incidence of dementia over 52 weeks in amnestic MCI patients treated with Ginkgo (16). Similarly, García-Alberca et al. found that Ginkgo in combination with acetylcholinesterase inhibitors improved cognitive and neuropsychiatric outcomes (28). While most studies have examined Ginkgo as an adjunct therapy, our study uniquely evaluates it in isolation, supported by biomarker evidence.

These results build upon our earlier study, which showed that the addition of Ginkgo to donepezil in amyloid PET–positive Alzheimer’s disease (AD) patients led to better cognitive outcomes and a larger decrease in MDS-Oaβ (20). However, in that study, Ginkgo was not administered independently, and all patients received donepezil due to ethical standards. This limited our ability to isolate Ginkgo’s effect. In contrast, the current study focused on MCI patients—a population where anti-dementia drugs are not reimbursed in Korea—allowing us to observe the standalone effects of Ginkgo in a real-world setting.

The cognitive preservation observed in the Ginkgo group is notable, especially considering that approximately 5–30% of amyloid PET–positive MCI cases convert to AD annually (4, 5). While the non-Ginkgo group showed mild decline in K-MMSE and modest functional worsening, the Ginkgo group not only maintained but slightly improved in both cognitive and functional outcome measures. These changes, though modest in magnitude, are clinically relevant given the context of early AD intervention, where even stabilization is a meaningful therapeutic target. The improvement in MDS-Oa*β* levels further supports the potential disease-modifying effect of Ginkgo. This plasma biomarker reflects the dynamic propensity of Aβ monomers to oligomerize, a process that is believed to play a critical pathogenic role in AD by disrupting synaptic function and triggering neuroinflammation. Traditional biomarkers like CSF Aβ42 and tau levels are static measures, whereas MDS-Oaβ offers insight into ongoing amyloidogenic activity. The observed reduction in MDS-Oaβ in the Ginkgo group may indicate a suppression of toxic oligomer formation.

Mechanistically, standardized extracts of *Ginkgo biloba* contain bioactive components including flavonol glycosides (e.g., quercetin, kaempferol, and isorhamnetin) and terpene lactones (ginkgolides and bilobalide), which provide distinct yet complementary protective effects against amyloid-beta (Aβ) pathology. Flavonol glycosides directly interact with Aβ peptides, disrupting the formation of β-sheet-rich oligomeric structures and inhibiting fibril nucleation, thus attenuating oligomer-induced synaptic toxicity. Additionally, their antioxidative activity mitigates oxidative stress-induced neuronal damage, a known accelerator of Aβ aggregation (8, 9). Ginkgolides, particularly ginkgolide B, have demonstrated potential inhibitory effects on β-secretase (BACE-1), the enzyme critically involved in amyloidogenic cleavage of amyloid precursor protein (APP), thereby reducing Aβ generation at an early stage. Bilobalide further contributes neuroprotective effects through mitochondrial stabilization and promotion of autophagy-mediated clearance of misfolded proteins (29, 30). Collectively, these pharmacological activities likely synergize to reduce Aβ oligomer formation and deposition, as reflected by decreased plasma MDS-Oaβ levels observed in our study. Importantly, these effects were achieved without the use of donepezil, rivastigmine, or memantine, highlighting the potential of Ginkgo as a monotherapy in the prodromal stage of AD.

The robustness of these findings is supported by two converging observations. First, our earlier add-on trial in amyloid-positive AD dementia showed that *Ginkgo biloba* produced a comparable magnitude of benefit when layered on top of donepezil, and that benefit tracked with a parallel reduction in plasma MDS-Oaβ levels (20). Second, in the present study, the non-Ginkgo group appeared to follow a clinical and biomarker trajectory similar to the natural course reported in longitudinal cohorts of amyloid-positive MCI: CDR-SB worsened over 12 months (4, 31), and MDS-Oaβ levels increased in a pattern consistent with previously reported biomarker trajectories in early-stage AD (27). The observation that the non-Ginkgo group showed clinical and biomarker trajectories similar to those reported in previous studies suggests that the likelihood of systematic bias or measurement artefact may be low and provides some support for the interpretation of stability observed in the Ginkgo group. Nonetheless, because our design is retrospective and unblinded, these signals should be viewed as hypothesis-generating rather than confirmatory.

The study also holds significance in the context of current therapeutic limitations. Recently approved anti-amyloid agents such as lecanemab and donanemab have shown promise in modifying disease progression. While direct comparisons must be approached with caution due to substantial methodological differences, nonetheless the cognitive and functional outcomes in the Ginkgo group appeared relatively stable, and this observation may be of interest in the context of findings from recent monoclonal antibody trials. For example, in the Clarity AD study of lecanemab, all participants exhibited some degree of cognitive decline over 12 months, with a mean increase of approximately 1.2 points in CDR-SB despite treatment (32). A similar pattern of worsening was observed in the TRAILBLAZER-ALZ 1 trial of donanemab (33). In our study, however, no mean change in CDR-SB was observed and slight improvement in MMSE was noted over a comparable period. Moreover, their high costs, intravenous administration requirements, and limited availability hinder widespread adoption, particularly in countries without full insurance coverage. In contrast, *Ginkgo biloba* is accessible, affordable, and orally administered, making it a pragmatic option for early intervention. Furthermore, the ethical and logistical challenges of conducting large, randomized trials in biomarker-confirmed MCI populations make real-world data increasingly valuable. Our study, based on registry-derived information and supported by biomarker assays, strengthens the internal validity of our findings.

Despite these promising results, several limitations must be acknowledged. First, the retrospective and non-randomized nature of this study inherently limits causal inference. Factors such as health behaviors, comorbidities, caregiver support, and other unmeasured variables might have influenced outcomes. Although we attempted to minimize these effects by comparing clinically similar groups, we did not apply formal statistical adjustments for baseline characteristics, and thus the possibility of residual confounding should be considered when interpreting between-group differences. Second, our sample size, while adequate for exploratory analysis, may not provide sufficient power to detect subtler subgroup effects or rare adverse events. Third, adherence to the assigned treatments was not objectively measured, although follow-up visits confirmed continued use in most cases. Fourth, while MDS-Oaβ is a promising biomarker, it should be acknowledged that MDS-Oaβ is not yet a globally standardized biomarker. To date, numerous peer-reviewed international studies have been published using MDS-Oaβ, demonstrating consistent analytic validity and substantial clinical utility in various contexts. Although its analytical performance can be sensitive to preanalytical factors—such as freeze–thaw cycles, sample handling, and storage duration—continuous efforts have been made to optimize and standardize these procedures. While there are currently no globally accepted regulatory standards or universal clinical thresholds for MDS-Oaβ, the method has already been approved by regulatory authorities in some countries (e.g., the Ministry of Food and Drug Safety, MFDS in Korea) and is actively utilized in clinical research. Thus, given its growing evidence base, MDS-Oaβ is emerging as a promising biomarker for Alzheimer’s disease, warranting further prospective validation studies. Lastly, we did not assess other potential biomarkers such as plasma p-tau or neurofilament light chain (NfL), which could provide additional insights into disease progression. Nonetheless, the findings of this study carry practical implications. In healthcare systems where early AD treatments are not accessible or reimbursed, *Ginkgo biloba* may represent a viable first-line option for biomarker-confirmed MCI. Its tolerability profile and multi-mechanistic action make it especially suitable for early intervention strategies. For clinicians, the availability of real-world biomarker data may also encourage more personalized and proactive management of MCI. Future research should aim to validate these findings through prospective randomized controlled trials, ideally incorporating multimodal biomarkers (e.g., tau PET, digital cognitive tests, and CSF markers). Additionally, studies comparing Ginkgo to emerging AD treatments in head-to-head designs could clarify its position in the evolving therapeutic landscape.

## Conclusion

This study provides preliminary but compelling evidence that *Ginkgo biloba* monotherapy may offer clinical and biological benefits in amyloid PET–positive MCI. By stabilizing cognitive performance and reducing amyloid oligomerization in plasma, Ginkgo may represent a cost-effective and accessible option for early-stage intervention in the Alzheimer’s continuum.

## Data Availability

The original contributions presented in the study are included in the article/supplementary material, further inquiries can be directed to the corresponding author.

## References

[1] Gulisano W Maugeri D Baltrons MA Fà M Amato A Palmeri A. Role of amyloid-beta and tau proteins in Alzheimer's disease: confuting the amyloid cascade. J Alzheimer's Dis. (2018) 64:S611–31. doi: 10.3233/JAD-179935 29865055 PMC8371153

[2] Jack CR Bennett DA Blennow K Carrillo MC Dunn B Haeberlein SB. NIA-AA research framework: toward a biological definition of Alzheimer’s disease. Alzheimers Dement. (2018) 14:535–62. doi: 10.1016/j.jalz.2018.02.018, PMID: 29653606 PMC5958625

[3] Ossenkoppele R van Berckel BN Prins ND. Amyloid imaging in prodromal Alzheimer's disease. Alzheimer's Res Ther. (2011) 3:26. doi: 10.1186/alzrt88, PMID: 21936965 PMC3218803

[4] Vos SJ Verhey F Frölich L Kornhuber J Wiltfang J Maier W. Prevalence and prognosis of Alzheimer’s disease at the mild cognitive impairment stage. Brain. (2015) 138:1327–38. doi: 10.1093/brain/awv029, PMID: 25693589 PMC5013930

[5] Chen Y Denny KG Harvey D Farias ST Mungas D DeCarli C. Progression from normal cognition to mild cognitive impairment in a diverse clinic-based and community-based elderly cohort. Alzheimers Dement. (2017) 13:399–405. doi: 10.1016/j.jalz.2016.07.151, PMID: 27590706 PMC5451154

[6] Kim Y Kang DW Kim GH Kim KW Kim HJ Na S. Clinical practice guidelines for dementia: recommendations for cholinesterase inhibitors and memantine. Dement Neurocogn Disord. (2025) 24:1–23. doi: 10.12779/dnd.2025.24.1.1, PMID: 39944527 PMC11813556

[7] Tabassum NE Das R Lami MS Islam F Abdel-Daim MM Hossain MS. *Ginkgo biloba*: a treasure of functional phytochemicals with multimedicinal applications. Evid Based Complement Alternat Med. (2022) 2022:8288818. doi: 10.1155/2022/8288818, PMID: 35265150 PMC8901348

[8] Pietri S Maurelli E Drieu K Culcasi M Manenti S Alviano F. Cardioprotective and anti-oxidant effects of the terpenoid constituents of *Ginkgo biloba* extract (EGb 761). J Mol Cell Cardiol. (1997) 29:733–42. 9140830 10.1006/jmcc.1996.0316

[9] Luo Y Smith JV Paramasivam V Butko P Khan I Pierce WM. Inhibition of amyloid-beta aggregation and caspase-3 activation by the *Ginkgo biloba* extract EGb 761. Proc Natl Acad Sci USA. (2002) 99:12197–202. doi: 10.1073/pnas.182425199 12213959 PMC129421

[10] Dodge HH Zitzelberger T Oken BS Howieson DB Kaye JA Cook DJ. A randomized placebo-controlled trial of *Ginkgo biloba* for the prevention of cognitive decline. Neurology. (2008) 70:1809–17. doi: 10.1212/01.wnl.0000303814.13509.db 18305231 PMC2639649

[11] DeKosky ST Williamson JD Fitzpatrick AL Kronmal RA Ives DG Saxton JA. *Ginkgo biloba* for prevention of dementia: a randomized controlled trial. JAMA. (2008) 300:2253–62. doi: 10.1001/jama.2008.683, PMID: 19017911 PMC2823569

[12] ScherrerBAndrieuSOussetPJColeyNVellasBRobertP. Analysing time to event data in dementia prevention trials: the example of the GuidAge study of EGb761. J Nutr Health Aging. (2016) 19:1009–11. doi: 10.1007/s12603-015-0661-226624212

[13] Vellas B Coley N Ousset PJ Berrut G Dartigues JF Dubois B. Long-term use of standardised Ginkgo biloba extract for the prevention of Alzheimer’s disease (GuidAge): a randomised placebo-controlled trial. Lancet Neurol. (2012) 11:851–9. doi: 10.1016/S1474-4422(12)70206-5, PMID: 22959217

[14] Oken BS Storzbach DM Kaye JA. The efficacy of *Ginkgo biloba* on cognitive function in Alzheimer disease. Arch Neurol. (1998) 55:1409–15. doi: 10.1001/archneur.55.11.1409, PMID: 9823823

[15] Birks J Grimley EJ. *Ginkgo biloba* for cognitive impairment and dementia. Cochrane Database Syst Rev. (2007) 2:CD003120. doi: 10.1002/14651858.CD003120.pub2 17443523

[16] Tian J Shi J Wei M Chen Y Zhang L Zhang Y. Chinese herbal medicine Qinggongshoutao for the treatment of amnestic mild cognitive impairment: a 52-week randomized controlled trial. Alzheimers Dement. (2019) 5:441–9. doi: 10.1016/j.trci.2019.03.001 PMC6732732 31517031

[17] Zhao MX Dong ZH Yu ZH Chen H Li XY Yang ZF. Effects of *Ginkgo biloba* extract in improving episodic memory of patients with mild cognitive impairment: a randomized controlled trial. Zhong Xi Yi Jie He Xue Bao. (2012) 10:628–34. doi: 10.3736/jcim20120605 22704410

[18] Gavrilova SI Preuss UW Wong JW Mihaylova A Kinoshita T Winblad B. Efficacy and safety of *Ginkgo biloba* extract EGb 761 in mild cognitive impairment with neuropsychiatric symptoms: a randomized, placebo-controlled, double-blind, multi-center trial. Int J Geriatr Psychiatry. (2014) 29:1087–95. doi: 10.1002/gps.4103 24633934

[19] Pagotto GLO Santos LMO Osman N Grassi-Oliveira R Barros VG Stutz BA. *Ginkgo biloba*: a leaf of hope in the fight against Alzheimer’s dementia: clinical trial systematic review. Antioxidants. (2024) 13:651. doi: 10.3390/antiox13060651 38929090 PMC11201198

[20] Yang Y Koo MS Kwak YT. Efficacy of *Ginkgo biloba* as an adjunct to donepezil in amyloid PET-positive Alzheimer’s patients. Front Neurol. (2025) 16:1563056. doi: 10.3389/fneur.2025.1563056, PMID: 40134691 PMC11932902

[21] Youn YC Kang S Suh J Kim HJ Park HJ Lee SH. Blood amyloid-β oligomerization associated with neurodegeneration of AD. Alzheimer's Res Ther. (2019) 10:40. doi: 10.1186/s13195-019-0499-7 PMC6511146 31077246

[22] Petersen RC Smith GE Waring SC Ivnik RJ Tangalos EG Kokmen E. Mild cognitive impairment: clinical characterization and outcome. Arch Neurol. (1999) 56:303–8. doi: 10.1001/archneur.56.3.303, PMID: 10190820

[23] Chin J Park J Yang SJ Kim YJ Lee DY Seo SW. Re-standardization of the Korean-instrumental activities of daily living (K-IADL): clinical usefulness for various neurodegenerative diseases. Dement Neurocogn Disord. (2018) 17:11–22. doi: 10.12779/dnd.2018.17.1.11 30906387 PMC6427997

[24] An SSA Lee BS Yu JS Kim JY Park JS Kim YH. Dynamic changes of oligomeric amyloid β levels in plasma induced by spiked synthetic Aβ42. Alzheimer's Res Ther. (2017) 9:86. doi: 10.1186/s13195-017-0310-6 29041968 PMC5645921

[25] Wang MJ Yi S Han JY Kim H Seo SW Park K. Oligomeric forms of amyloid-beta protein in plasma as a potential blood-based biomarker for AD. Alzheimer's Res Ther. (2017) 9:98. doi: 10.1186/s13195-017-0324-0 29246249 PMC5732503

[26] Meng X Li T Wang X Lv X Sun Z Zhang J. Association between increased levels of amyloid-β oligomers in plasma and episodic memory loss in Alzheimer’s disease. Alzheimer's Res Ther. (2019) 11:89. doi: 10.1186/s13195-019-0535-7, PMID: 31651358 PMC6814096

[27] Nah EH Cho S Park H Kim J Lee BH Lee J. Reference interval and the role of plasma oligomeric beta amyloid in screening of risk groups for cognitive dysfunction at health checkups. J Clin Lab Anal. (2021) 35:e23933. doi: 10.1002/jcla.23933 34342379 PMC8418474

[28] García-Alberca JM Gris E Mendoza S Martín A Mateo I Sevillano CM. Combined treatment with *Ginkgo biloba* extract EGb 761 plus acetylcholinesterase inhibitors improved cognitive function and neuropsychiatric symptoms in patients with mild cognitive impairment. Alzheimers Dement. (2022) 8:e12338. doi: 10.1002/trc2.12338 PMC9345397 35929002

[29] Wu Y Wu Z Butko P Christen Y Lambert MP Klein WL. Amyloid-beta-induced pathological behaviors are suppressed by *Ginkgo biloba* extract EGb 761 and ginkgolides in transgenic *Caenorhabditis elegans*. J Neurosci. (2006) 26:13102–13. doi: 10.1523/JNEUROSCI.3448-06.2006, PMID: 17167099 PMC6674971

[30] Wan W Zhang C Danielsen M Li Q Chen W Chan Y. EGb 761 improves cognitive function and regulates inflammatory responses in the APP/PS1 mouse. Exp Gerontol. (2016) 81:92–100. doi: 10.1016/j.exger.2016.05.007, PMID: 27220811

[31] Aisen PS Donohue MC Raman R Rafii MS Petersen RC Weiner MW. The Alzheimer's disease neuroimaging initiative clinical Core. Alzheimers Dement. (2024) 20:7361–8. doi: 10.1002/alz.14167 39136045 PMC11485391

[32] van Dyck CH Swanson CJ Aisen PS Bateman RJ Chen C Gee M. Lecanemab in early Alzheimer’s disease. N Engl J Med. (2023) 388:9–21. doi: 10.1056/NEJMoa2212948, PMID: 36449413

[33] Mintun MA Lo AC Duggan Evans C Wessels AM Ardayfio P Andersen SW. Donanemab in early Alzheimer’s disease. Lancet Neurol. (2021) 20:412–22. doi: 10.1056/NEJMoa2100708 33720637

